# Overview of chest involvement at computed tomography in children with coronavirus disease 2019 (COVID-19)

**DOI:** 10.1007/s00247-020-04826-7

**Published:** 2020-10-21

**Authors:** Xuehua Peng, Yu Guo, Han Xiao, Wei Xia, Aiguo Zhai, Baiqi Zhu, Wenhan Zhang, Jianbo Shao

**Affiliations:** 1 Department of Imaging Center, Wuhan Children’s Hospital (Wuhan Maternal and Child Healthcare Hospital), Tongji Medical College, Huazhong University of Science & Technology, No. 100 Hong Kong Road, Jiang’an District, Wuhan, 430000 China; 2 Institute of Women and Children’s Healthcare, Wuhan Children’s Hospital (Wuhan Maternal and Child Healthcare Hospital), Tongji Medical College, Huazhong University of Science & Technology, No. 100 Hong Kong Road, Jiang’an District, Wuhan, 430000 China

**Keywords:** Chest, Children, Computed tomography, COVID-19, Lungs, SARS-CoV-2

## Abstract

**Background:**

Chest computed tomography (CT) findings in children with coronavirus disease 2019 (COVID-19) have been rarely reported in a comprehensive and systematic manner.

**Objective:**

We investigated the chest CT findings in children with COVID-19, and explored the differences in these findings between symptomatic patients and asymptomatic patients.

**Materials and methods:**

Demographic findings, clinical characteristics, duration of hospital stay and viral shedding, and chest CT findings in 201 children infected with severe acute respiratory syndrome coronavirus-2 (SARS-CoV-2) were retrospectively analyzed from January 15 to March 20, 2020, and divided into two groups: symptomatic group (*n*=136) and asymptomatic group (*n*=65). Chi-square test and Student’s *t*-test were used for statistical analysis.

**Results:**

Symptomatic patients were mainly young children ≤3 years old (54/63, 86%),while asymptomatic patients were mainly children ≥ 6 years old (51/111, 46%). Fever (41%) and cough (41%) were the most common symptoms. Overall, 119/201 (59%) patients had chest CT findings, and symptomatic patients accounted for 82% (98/119). The CT findings presented as bilateral multiple lesions (60/119, 50.4%), ground-glass opacities (83/119, 70%) and/or consolidation (44/119, 37%) with a peripheral and subpleural distribution (62/83, 75%). Fifteen of 87 (7.2%) patients with lung lesions showed complete lesion absorption, and 42/87 (48%) improved within a mean of 9.1 (standard deviation [SD] 3.2) days. The mean duration of viral shedding was 8.7 (SD 4.9) days. Pleural effusion was very rare. No lymphadenopathy was found in either group.

**Conclusion:**

Symptoms associated with pulmonary involvement were most common in infants and young children. The lung lesions of most patients were absorbed and improved in about 9 days.

## Introduction

Since the initial outbreak of severe acute respiratory syndrome coronavirus-2 (SARS-CoV-2) which causes coronavirus disease 2019 (COVID-19) in December 2019, the pandemic has critically affected people’s work and quality of life. Several countries are still struggling to control the number of infected cases, while others, like China, have managed to reduce the numbers. Children worldwide have also been infected with COVID-19.

During the initial stage of the outbreak, COVID-19 was first noticed by medical staff because of the unique pulmonary imaging manifestations in adults owing to the respiratory symptoms. However, given the lack of nucleic acid detection kits, a quicker identification of patients with suspected COVID-19 was made possible using a dedicated computed tomography (CT) scanner [1, 2]. CT was also the main diagnostic method followed by “Pneumonia diagnosis and treatment guideline for SARS-CoV-2 infection (trial version 5)” issued by the National Health Commission of the People’s Republic of China. CT findings play an important role in detecting lung abnormalities and facilitate the early identification of the disease.

Ground-glass opacity is the main finding in the early stages of COVID-19. It is easy to miss this lesion on radiography, and it is also difficult to evaluate the distribution, scope and progression of the lesion. The pulmonary manifestations of COVID-19 in pediatric pneumonia are less severe than those of adults, the extent of involvement is small, and it is more difficult to identify the pulmonary lesions by radiography. The current report also focuses on the analysis of the imaging manifestations of COVID-19 pneumonia and compares to imaging manifestations of COVID-19 pneumonia in adults [3, 4].

To the best of our knowledge, there is a lack of comprehensive analysis from the clinical perspective of pulmonary involvement at different ages. Furthermore, inadequate attention has been paid to clinical research on asymptomatic subjects, especially children. As Wuhan was the center of the epidemic in China, ours was the only designated hospital to manage pediatric COVID-19 cases, including confirmed and suspected patients in Wuhan City. Our hospital integrated isolation and treatment of pediatric patients and carried out district management for suspected or confirmed infections. Therefore, we obtained large samples of clinical and imaging data of pediatric COVID-19 cases. The main purpose of this study was to report the chest CT findings of COVID-19 infection in children. We conducted a comprehensive analysis of pulmonary involvement in children with COVID-19 at different ages, and objectively evaluated the rational and standardized application of lung CT in the diagnosis of pediatric COVID-19 pneumonia.

## Materials and methods

### Patients

This retrospective study was supported by the Medical Ethics Committee of Wuhan Children’s Hospital (Wuhan Maternal and Child Healthcare Hospital) (IEC-2020R003-e01) and written informed consent was waived for emerging infectious diseases. This work was funded by the Special Project for Emergency of the Huazhong University of Science & Technology.

From January 15 to March 20, 2020, we studied the medical records and admission chest CT findings from the electronic medical records of our hospital information system and PACS, respectively, of 211 pediatric patients. All children had a diagnosis of COVID-19 based on a positive real-time reverse transcription polymerase chain reaction (RT-PCR) test and epidemic history. We excluded 10 patients with more than 1 pathogen or diagnosis, including 7 with mycoplasma infection, 1 with streptococcus infection, 1 with acute leukemia and 1 with intussusception followed by operation. Of the 201 patients (118 boys and 83 girls; median age: 6 years [range: 3 h to 15 years]), 136 were symptomatic and 65 were asymptomatic. Asymptomatic patients were those with positive RT-PCR results but no clinical symptoms with or without abnormal findings on chest CT or abnormal laboratory tests.

Radiologic assessments included chest CT without contrast. Two radiologists (X.P. and A.Z., with 15 and 10 years of experience in radiology, respectively) independently reviewed the chest CT images. Major disagreement between the two reviewers was resolved by consultation with a third reviewer (J.S., with 35 years of experience in radiology). Only final decisions reached by consensus are reported. The radiologists described main CT features defined by the Fleischner Society (ground-glass opacity, consolidation, interlobular septal thickening, halo sign, crazy-paving pattern, nodules) [5]. Additionally, lesion distribution and location, pleural effusion and lymphadenopathy were also evaluated. The data of 87 children with chest CT follow-up within 15 days were collected.

We compared the hospitalization period and the median duration of viral shedding between the two groups. Patient discharge criteria for pediatric patients in this hospital were normal body temperature for 3 days, two negative RT-PCR results at 24-h intervals and resolution of all clinical symptoms [3]. The same criteria were applied for asymptomatic children. During hospitalization, we observed whether they became symptomatic. Even if they were asymptomatic throughout, they had to have two negative RT-PCR results at 24-h intervals before being discharged from the hospital. The hospitalization period was based on the World Health Organization (WHO) guidelines [6].

A RT-PCR test to detect SARS-CoV-2 was performed every 2–7 days (mean: 3 days). The duration of viral shedding was considered as time from confirmation date to the day before the first of two consecutive negative results of RT-PCR tests.

We also divided the 201 children into lung lesion positive (119 cases) and lung lesion negative (82 cases) groups and compared the onset age, body temperature and hospitalization period of the two groups. We also analyzed the onset age and the time from symptoms to definite diagnosis and from symptoms to CT examination for children with symptoms and lung lesions.

### Chest CT scan protocol

All patients were imaged on a Siemens SOMATOM Definition AS128 (Siemens Healthineers, Erlangen, Germany) or on a GE Optima CT 660 (GE Medical Systems, Milwaukee, WI). All scans were obtained with the patient in the supine position without contrast. The scanning range covered lung apex to diaphragm on the axial plane taken under free breathing. Scanning parameters were as follows: tube voltage=120 kV, tube current modulation 60–120 mAs (mean±standard deviation [SD]: 67.55±11.94 mAs) on Siemens SOMATOM Definition AS128 and 10–31 mAs (mean±SD: 17.78±7.17 mAs) on GE Optima CT 660, pitch=1.375–1.4, matrix=512×512, slice thickness=10 mm. All images were then reconstructed with a slice thickness of 0.625–1.0 mm. Nine children (1–3 years old) who did not cooperate received oral 10% chloral hydrate (0.5 mL/kg body mass) sedation before CT examination. A sick child with breathing problems was not sedated, but the clinician accompanied the child to complete the examination.

### Real-time reverse transcription polymerase chain reaction assay for SARS-CoV-2

Nasopharyngeal swabs from children younger than 2 years of age and throat swabs from children 2 years or older were obtained to detect SAR-CoV-2 ribonucleic acid (RNA). RNA from the respiratory specimens was extracted with the High Pure Viral Nucleic Acid Kit (Zhongzhi, Wuhan, China). The extracted nucleic acids were tested for SAR-CoV-2 using RT-PCR assay [7].

### Statistical analysis

Statistical analyses were done using the SPSS software (version 25; IBM, Armonk, NY). The clinical characteristics, CT findings, and chest involvement in different age groups were compared using chi-square test between the symptomatic group and the asymptomatic group, and laboratory results were compared using the Student’s *t*-test. *P*<0.05 was considered statistically significant.

## Results

### Demographic and clinical characteristics (Table 1, Fig. 1)

Among 201 pediatric cases of confirmed COVID-19, 118 (58.7%) were boys. There was no difference with respect to gender between the two groups, of which 70.3% (83/118) had symptoms. SARS-CoV-2 can infect all age groups; of the 55% (111/201) of children ≥6 years old, 54.1% (60/111) were symptomatic and 45.9% (51/111) were asymptomatic. When comparing patients with a definite history of close contact with confirmed COVID-19 family members, 73.6% (148/201) had such a history of contact, while 16.4% (33/201) patients had suspected contact. Clinical manifestations were as follows: fever (41.3% [83/201]) defined as an axillary temperature over 37.5 °C and cough (40.8% [82/201]) were the most common symptoms, followed by phlegm in the throat (9.5% [19/201]), rhinorrhea (7.4% [15/201]), fatigue (7.0% [14/201]), diarrhea (4.5% [9/201]), tachypnea (3.5% [7/201]), sneezing (3.0% [6/201]), vomiting (3.0% [6/201]), nasal obstruction (2.5% [5/201]) and muscle soreness (1.0% [2/201]) (Table 1). There were significant differences in axillary temperature between the asymptomatic group (36.5±0.4) and the symptomatic group (37.2±1.1). Leukopenia and lymphocyte counts decreased by 8.0% (16/201) and 4.5% (9/201), respectively.

**Table 1:** Pediatric patient demographics (all RT-PCR-confirmed COVID-19 positive)

Clinical characteristic	All patients (*n*=201)	Symptomatic group (*n*=136)	Asymptomatic group (*n*=65)	*P*
Gender				
boys	118/201 (58.7%)	83/118 (70.3%)	35/118 (29.7%)	0.33
girls	83/201 (41.3%)	53/83 (63.9%)	30/83 (36.1%)	
Age				
<1 month	7/201 (3.5%)	6/7 (85.7%)	1/7 (14.3%)	
1 month–1 year	34/201 (16.9%)	32/34 (94.1%)	2/34 (5.9%)	
1–3 years	22/201 (11.0%)	16/22 (72.7%)	6/22 (27.3%)	
3–6 years	27/201 (13.4%)	22/27 (81.5%)	5/27 (18.5%)	
6–12 years	76/201 (37.8%)	41/76 (53.9%)	35/76 (46.1%)	
≥12 years	35/201 (17.4%)	19/35 (54.3%)	16/35 (45.7%)	
median	6 years	4 years	7 years	
range	3 h–15 years	3 h–15 years	3 h–15 years	
Contact history				
family cluster	148/201 (73.6%)	92/148 (62.2%)	56/148 (37.8%)	
suspected contact	33/201 (16.4%)	26/33 (78.8%)	7/33 (21.2%)	
uncertain	20/201 (10.0%)	18/20 (90%)	2/20 (10.0%)	
Symptoms				
fever	83/201 (41.3%)	83/136 (61.0%)		
cough	82/201 (40.8%)	82/136 (60.3%)		
phlegm in the throat	19/201 (9.5%)	19/136 (14.0%)		
rhinorrhea	15/201 (7.4%)	15/136 (11.0%)		
fatigue	14/201 (7.0%)	14/136 (10.3%)		
diarrhea	9/201 (4.5%)	9/136 (6.6%)		
tachypnea	7/201 (3.5%)	7/136 (5.1%)		
sneezing	6/201 (3.0%)	6/136 (4.4%)		
vomiting	6/201 (3.0%)	6/136 (4.4%)		
nasal obstruction	5/201 (2.5%)	5/136 (3.7%)		
muscle soreness	2/201 (1.0%)	2/136 (1.5%)		
Laboratory results^a^				
Leukopenia	16/201 (8.0%)	15/16 (93.8%)	1/16 (6.2%)	
Decreased lymphocyte count	9/201 (4.5%)	8/9 (88.9%)	1/9 (11.1%)	
Temperature (°C)				
Axillary temperature	36.97±0.9	37.2±1.1	36.5±0.4	<0.0001
Duration of viral shedding (mean days±SD)	8.7±4.9	8.5±4.9	9.2±5.1	0.26
Duration of hospitalization (mean days±SD**)**	13.2±5.9	13.6±6.2	12.6±5.1	0.41

*COVID-19* coronavirus disease 2019, *RT-PCR* reverse transcription polymerase chain reaction, *SD* standard deviation

^a^ Normal ranges: white blood cell count 5.5–12.0 × 10^9^/L; lymphocyte count 1.2–6.0 × 10^9^/L

**Fig. 1:**
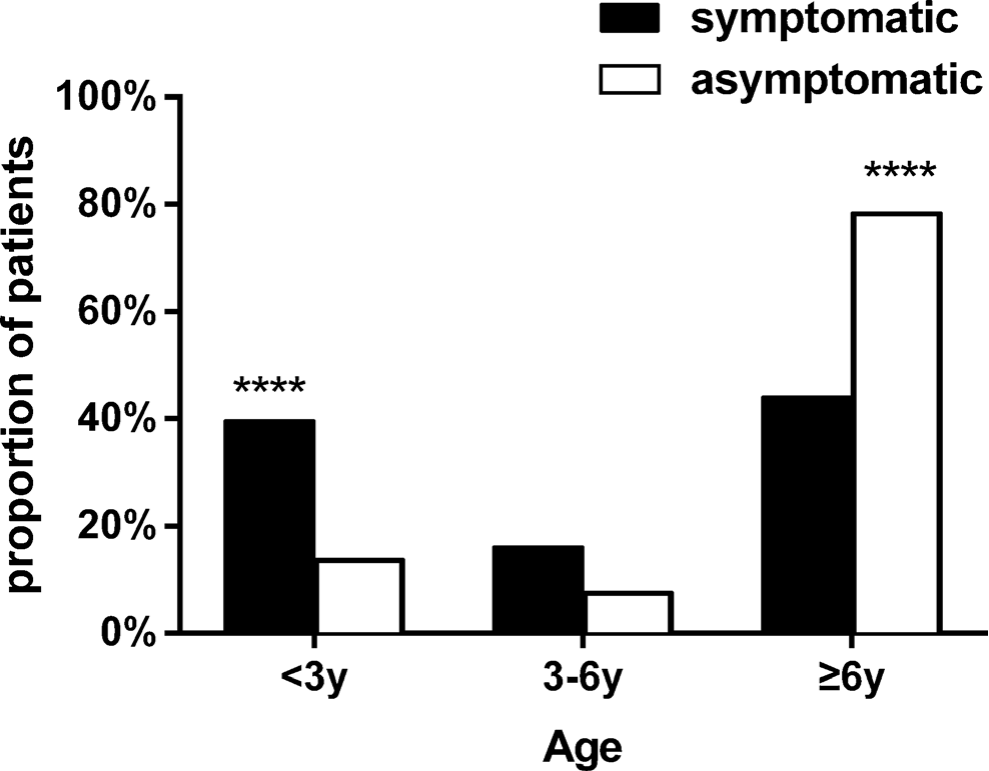
The age distribution of COVID-19 pediatric patients. Comparison of proportion of population with different age distribution in the symptomatic group and the asymptomatic group. There were significant differences in the group younger than 3 years old and the group older than 6 years. **** *P*<0.0001

### Chest CT findings (Tables 2 and 3, Figs. 2, 3, 4, 5, 6 and 7)

All 201 children underwent lung CT scan with 59.2% (119/201) showing positive CT findings, 82.4% (98/119) in the symptomatic group and 17.6% (21/119) in the asymptomatic group. The lung CT manifestations of the two groups were significantly different, and the differences between the two groups were more obvious for children >6 years old. Among the pulmonary positive cases, single lesions accounted for 24.4% (29/119), multiple lesions accounted for 75.6% (90/119) and multiple lesions in both lungs accounted for 50.4% (60/119). Multiple lesions accounted for 81.6% (80/119) in the symptomatic group, and unilateral lung lesions accounted for 76.2% (16/21) in the asymptomatic group with significant intergroup statistical differences. The main imaging findings were ground-glass opacities (69.7% [83/119]) and consolidation (37.0% [44/119]), of which ground-glass opacities associated with peripheral and subpleural areas accounted for 74.7% (62/83) and ground-glass opacities with crazy paving stone sign accounted for 3.6% (3/83). The rate of consolidation with halos was 47.7% (21/44). Lesions with interlobular septal thickening accounted for 4.2% (5/119). Striated shadows and small nodule shadows (≤5 mm) accounted for 11.8% (14/119) and 5.0% (6/119), respectively. Overall, the data of 87 children who were followed up within 15 days were collected, and the interval between the two examinations was 8.7±3.0 days on average. The following CT findings were observed after follow-up: complete lesion absorption (17.2%, 15/87); lesion improvement (48.3%, 42/87) with slight ground-glass opacity remaining locally; lesion progression (23%, 20/87); no change in lesion (3.4%, 3/87); and remaining fibrous cords (6.9%, 6/87). There were no changes at follow-up with respect to streak shadow (five cases) and small nodule shadow (two cases) detected in the first chest CT. Complete lesion absorption and lesion improvement was identified within a mean of 9.1±3.2 days. Pleural effusion occurred in one critically ill patient as the disease progressed. No enlargement of mediastinal or hilar lymph node was found in either group.

**Table 2:** CT findings of COVID-19 pediatric patients

Chest findings	All patients (*n*=201)	Symptomatic group (*n*=136)	Asymptomatic group (*n*=65)	*P*
Negative	82/201 (40.8%)	38/136 (27.9%)	44/65 (67.7%)	0.01
Positive	119/201 (59.2%)	98/136 (72.1%)	21/65 (32.3%)	
Distribution				
A lesion in unilateral lung	29/119 (24.4%)	18/98 (18.4%)	11/21 (52.4%)	
Multiple lesions in unilateral lung	30/119 (25.2%)	25/98 (25.5%)	5/21 (23.8%)	
Multiple lesions in bilateral lung	60/119 (50.4%)	55/98 (56.1%)	5/21 (23.8%)	
Characteristic				
Ground-glass opacity	83/119 (69.7%)	70/98 (71.4%)	13/21 (61.9%)	
Peripheral and subpleural area	62/83 (74.7%)	55/70 (78.6%)	7/13 (53.8%)	
Crazy paving pattern	3/83 (3.6%)	2/70 (2.9%)	1/13 (7.7%)	
Consolidation	44/119 (37.0%)	38/98 (38.8%)	6/21 (28.6%)	
Halo sign	21/44 (47.7%)	19/38 (50.0%)	2/6 (33.3%)	
Peripheral and subpleural area	36/44 (81.8%)	31/36 (86.1%)	5/6 (83.3%)	
Nodule (≤5 mm)	6/119 (5.0%)	3/98 (3.1%)	3/21 (14.3%)	
Interlobular septal thickening	5/119 (4.2%)	5/98 (5.1%)	0/21(0.0)	
Streaky opacities	14/119 (11.8%)	8/98 (8.2%)	6/21 (28.6%)	
Pleural effusion	1/119 (0.8%)	1/98 (1.0%)	0/21(0.0)	

Comparison of lesion involvement range (unilateral and bilateral) and number (mono and multiple). There were significant differences between both groups with *P*<0.007 and *P*<0.001, respectively. *COVID-19* coronavirus disease 2019

**Table 3:** Overview of CT findings of lung involvement in both groups

Chest lesion	All patients (*n*=201)	Symptomatic group (*n*=136)		Asymptomatic group (*n*=65)		*P*
		Positive	Negative	Positive	Negative	
<1 month	7/201 (3.5%)	4/136 (2.9%)	2/136 (1.5%)	0/65 (0%)	1/65 (1.5%)	
1 month–1 year	34/201 (16.9%)	28/136 (20.6%)	4/136 (3.0%)	1/65 (1.5%)	1/65 (1.5%)	
1–3 years	22/201 (10.9%)	10/136 (7.4%)	6/136 (4.4%)	2/65 (3.1%)	4/65 (6.2%)	
3–6 years	27/201 (13.4%)	13/136 (9.6%)	9/136 (6.6%)	1/65 (1.5%)	4/65 (6.2%)	
6–12 years	76/201 (37.8%)	28/136 (20.6%)	12/136 (8.8%)	14/65 (21.5%)	22/65 (33.8%)	0.006
≥12 years	35/201 (17.4%)	15/136 (11.0%)	5/136 (3.7%)	3/65 (4.6%)	12/65 (18.5%)	0.001

Any chest CT finding = positive, no CT finding = negative

**Fig. 2:**
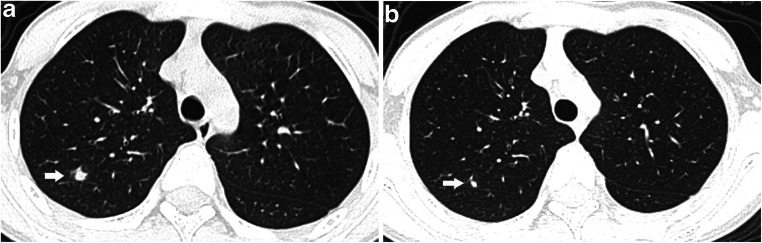
A 10-year-old boy with COVID-19, with fever and a history of exposure. **a** Axial thin-section non-contrast CT shows nodule shadow in the posterior segment of the right upper lobe (*arrow*). **b** Follow-up CT image shows the lesion is markedly reduced after 21 days (*arrow*)

**Fig. 3:**
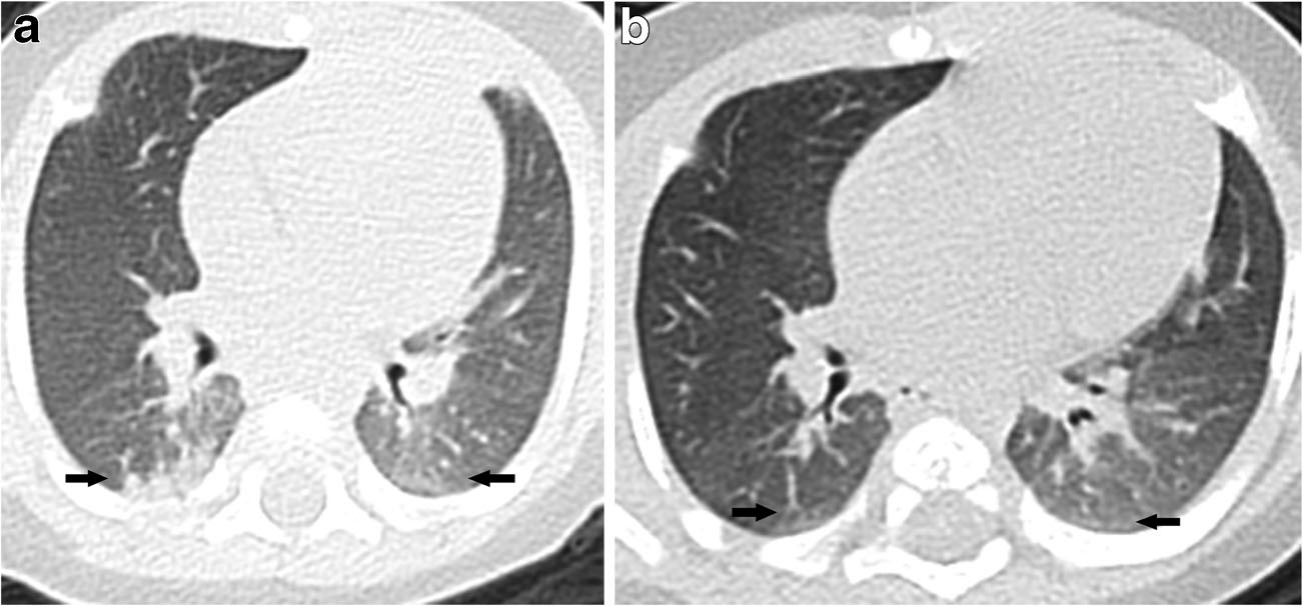
A 2-month-old symptomatic girl with COVID-19 and a history of definite contact presented with paroxysmal cough and rhinorrhea. **a** Axial thin-section non-contrast CT shows ground-glass opacities and consolidation in the subpleural zone of both lower lobes (*arrows*). **b** A follow-up CT image shows the lesions are markedly absorbed after 1 month (*arrows*)

**Fig. 4:**
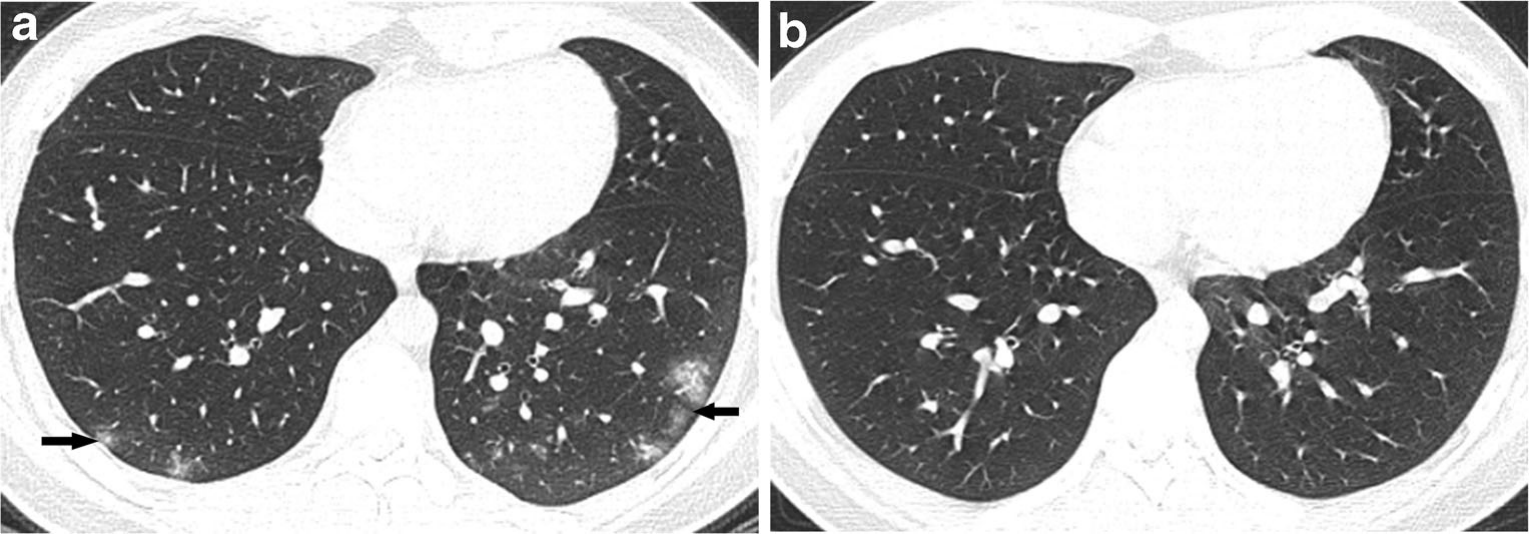
A 15-year-old symptomatic boy with COVID-19 and a history of definite contact presented with fever, cough, muscular soreness and fatigue. **a** Axial thin-section non-contrast CT shows scattered patchy ground-glass opacities in both lower lobes with a peripheral distribution (*arrows*). **b** A follow-up CT image shows normal lung after 1 month

**Fig. 5:**
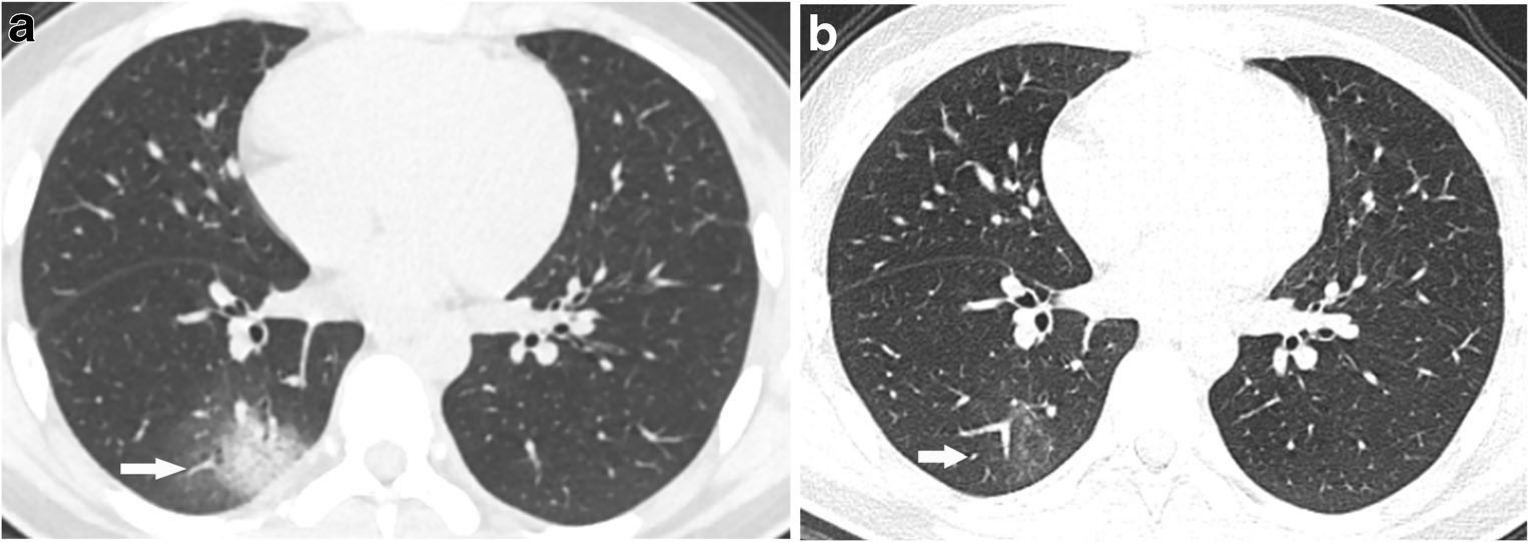
A 15-year-old asymptomatic boy with COVID-19 and a history of definite contact. **a** Axial thin-section non-contrast CT shows a subpleural rounded area of consolidation surrounded by ground glass, consistent with the halo sign and situated at the right lower lobe (*arrow*). **b** A follow-up CT image shows light ground-glass opacities after 9 days (*arrow*)

**Fig. 6:**
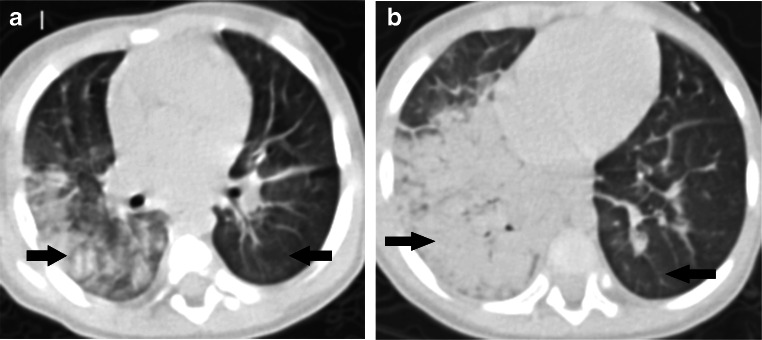
A 1-year old critically ill boy with COVID-19 with diarrhea, fever and a history of exposure. **a, b** Axial thin-section non-contrast CT images show diffuse ground-glass opacities bilaterally, extensive consolidation at the right lung and reticulation bilaterally (*arrows*)

**Fig. 7:**
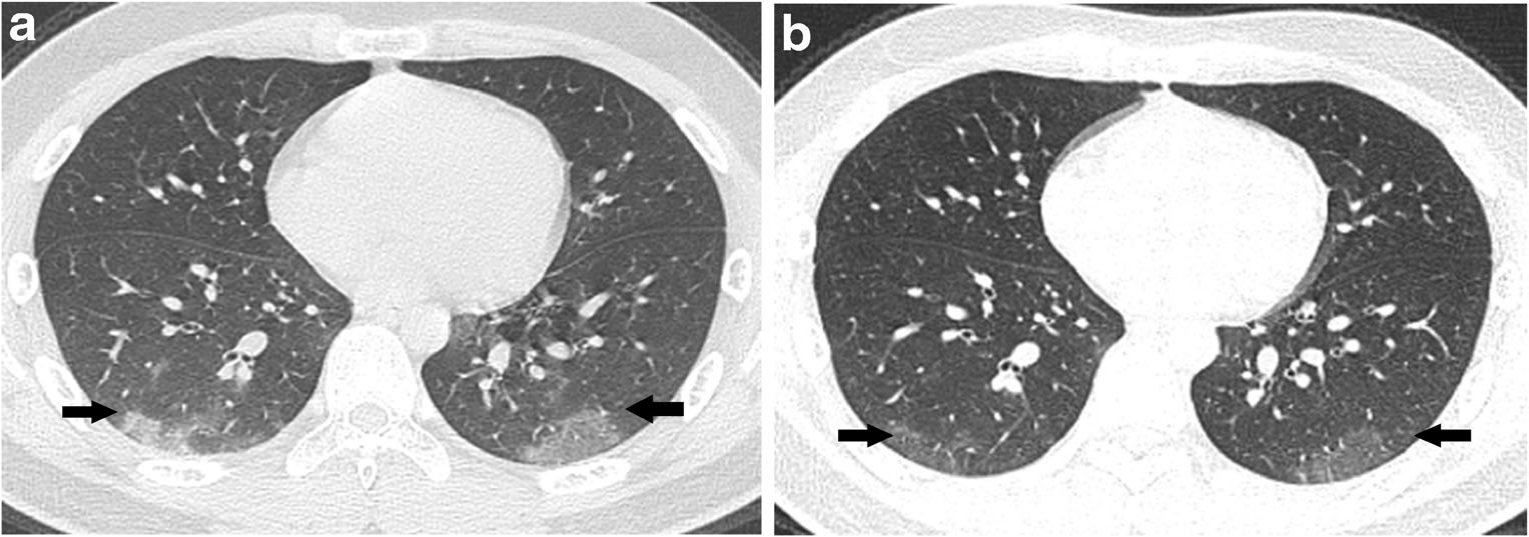
A 13-year-old symptomatic boy with COVID-19 with fever, cough and a history of exposure. **a** Axial thin-section non-contrast CT shows patchy peripheral ground-glass opacities in both lower lobes (*arrows*). **b** A follow-up CT image shows light ground-glass opacities after 5 days (*arrows*)

### Hospitalization (Table 4)

The duration of hospitalization ranged from 4 to 34 days, with a mean of 13.2±5.9 days. The hospitalization periods of the symptomatic and asymptomatic groups were 13.6±6.2 and 12.6±5.1 days, respectively, and the mean duration of viral shedding was 8.5±4.9 and 9.2±5.1 days, respectively. There was no statistical difference in hospitalization duration and nucleic acid negative conversion. The average ages of lung positive and negative groups were 5.7±4.8 years and 7.0±4.2 years, respectively, and the average body temperature was 37.1±1.0 °C and 36.8±0.9 °C, respectively. The average hospital stay was 12.8±6.0 days and 13.1±5.9 days, respectively, with statistical differences in age and body temperature between the lung positive and negative groups. About 48.8% (98/201) of the children with symptoms and pulmonary CT abnormalities were 5.1±4.9 years old. The median duration from the first symptom to laboratory confirmation was 6.3±4.5 days and to CT examination was 4.8±5.1 days, and the time interval between CT manifestation abnormality and nucleic acid examination was 3.2±2.9 days.

**Table 4:** Differences in age, temperature and hospital stays between the two groups

Lung findings	Positive findings	Negative findings	*P*
	(*n*=119)	(*n*=82)	
	Mean±SD	Mean±SD	
Age (year)	5.7±4.8	7.0±4.2	0.039
Temperature (°C)	37.1±1.0	36.8±0.9	0.035
Hospital stays (day)	12.8±6.0	13.1±5.9	0.704

*SD* standard deviation

## Discussion

Thus far, extensive data have shown that the clinical presentations in patients with COVID-19 can vary from asymptomatic infection to severe disease and even death. The death rate of adults with severe illness, especially those who are middle-aged or elderly, is further increased if they have underlying comorbidities [8, 9]. Overall, the number of confirmed pediatric cases has been very low, with most patients being asymptomatic or having mild or moderate infections [6, 10–13]. It remains unclear whether children with COVID-19 are contagious. At the initial stage of the infection, symptomatic patients are a considerable cause for concern. With the spread of the pandemic, family or community-based events, especially those harboring some asymptomatic infected patients, are a source of contagion and a genuine cause for concern [7, 14, 15]. The relative transmissibility of asymptomatic cases could be significantly smaller than that of symptomatic cases [15]. Studies have shown that asymptomatic infections are more common in populations of young and middle-aged individuals without underlying diseases [16, 17]. Asymptomatic infections in children have only been recently reported [10]. The asymptomatic group accounted for 32.3% (65/201) of patients in our study, slightly more than that reported in a previous report [17]. The criteria of defining no symptoms were different. Some studies excluded patients with lung lesions [13]. There was no gender-based difference, but significant differences in age composition between the two groups were found. Children ≤3 years old are more likely to show symptoms, while children ≥6 years old had a greater tendency to be asymptomatic (*P*<0.0001). The mean age of the symptomatic group was 4 years old, which was less than that of the asymptomatic group (mean: 7 years old). Fever and cough were the main clinical symptoms, accounting for 61.0% (83/136) and 60.3% (82/136) of patients, respectively, the same as in adults. The mean body temperature of the symptomatic group was 37.2±1.1 °C, rarely showing high fever. Critical illness was very rare among children. There was only one critically ill patient in our study, who fully recovered and was discharged from our hospital. Infected children had declined leukopenia and lymphocyte in 8.0% (16/201) and 4.5% (9/201) of cases, respectively. The rate was lower than that in reports of adult cases [8, 9].

COVID-19 pneumonia can occur in unilateral or bilateral lung with single or multiple lesions, typically with peripheral and subpleural distribution and bilateral multifocal lower-lobe predominance [1–3]. We got the same results. The bilateral multiple lesions accounted for 50.4% (60/119) of cases. Bilateral multiple lesions were obviously dominant in the symptomatic group (56.1% [55/98]) [6], while unilateral lesions were obviously dominant in the asymptomatic group (76.2% [16/21]), especially unilateral single lesions (52.4% [11/21]). Negative CT findings were found in 27.9% (38/136) of the symptomatic group and 67.7% (44/65) of the asymptomatic group. These results were different from those of other studies [18]: (1) Our asymptomatic patients did not show any symptoms until discharge, while it was reported that all patients showed abnormalities of CT findings at admission, and 27.6% patients presented symptoms after admission, and (2) the rate of bilateral lesions was lower (23.8% vs. 41.4%) and that of unilateral lesions was higher (76.2% vs. 58.6%) than those in adult reports. There were significant statistical differences in lesion involvement range (*P*<0.007) and number (*P*<0.001) between the two groups. CT findings and location were largely the same.

Lung lesions mainly consisted of ground-glass opacities (69.7% [83/119]) and consolidation (37.0% [44/119]), and 82.4% (98/119) of lesions showed peripheral and subpleural distribution, rarely with crazy paving pattern and interlobular septal thickening. Consolidation with the halo sign was seen in 47.7% (21/44) of patients. The lesions presented as various shapes including patchy opacities, linear opacities, nodular shadow, rounded morphology pattern and lobar airspace consolidation. The findings were closely related with the pathophysiological basis and lesion progression [18].

Chest CT was reexamined within 15 days of follow-up. The mean time interval between two chest CT scans was 8.7±3.0 days, and 17.2% (15/87) of them showed complete resolution of findings. The main findings were ground-glass opacities; even if multiple subsegments were involved, the patch shadow was small and scattered. Resolution rate of lesions was 48.3% (42/87), of which 52.4% (22/42) of cases showed obvious local residual weak ground-glass opacity or uneven local inflation. Residual fibrous cords with blurred boundaries were seen in 14.3% (6/42) of cases, but one case with residual fibrous cords of lung showed complete disappearance after 70 days of reexamination.

We speculated that the residual fibrous cords of lung may be absorbed with enough time. The focus in recovery has two main characteristics: the proportion of lung lobes and segments involved, and small patch shadow. There were 23.0% (20/87) of patients with lesion progression. We speculated that it was the development process of the lesion itself.

A few of these children were followed for a short period of time, within 5 days, and different children might have different reactions to the virus. No obvious changes were found in three cases. The streak shadow (five cases) and small nodular shadow (two cases) found on the initial chest CT showed no change during the follow-up. Although there were fiber streak shadow residues in the cases we followed, the mean time from symptom onset to the first CT examination was 4.8±5.1 days. Most lesions in the acute stage were in the initial stage of absorption and were fully absorbed within 9 days. Therefore, we believe that the first streak shadow and small nodular shadow with clear boundaries of the lung, which are occasionally seen during the examination, are not caused by SARS-CoV-2. This is different from the changes seen after COVID-19 in adults [19].

No enlargement of mediastinal or hilar lymph node was found. Pleural effusion occurred only in one critically ill patient as the disease progressed (0.5% [1/201]).

The incidence of positive findings in the chest accounted for 59.2% (119/201) of cases, far lower than that in adults (86.2–96.6%) [1, 8, 9]. About 90–100% of MERS patients and 60–100% of SARS patients had reported chest CT abnormalities [20]. The incidence of the negative findings in the chest accounted for 42% (85/201) in children, and there were significant differences compared to that (0.12% [126/1,014]) in adults [1]. Positive findings were mainly seen in the symptomatic group (70% [95/136]), while negative findings were seen in the asymptomatic group (68% [44/65]). There was a significant statistical difference between the two groups, suggesting that the incidence of lung abnormalities in asymptomatic children is relatively low.

We carried out statistical analysis of lung involvement in the two groups at different ages and found that most asymptomatic children with negative findings in chest CT were >6 years old. Children <3 years old showed clinical symptoms and the rate of incidence of pulmonary abnormalities was high, which has obvious statistical significance. We also divided the children into groups according to negative and positive lung findings, which were statistically significant with respect to age and body temperature. The average age of children with positive lung findings was lower than that of children with negative lung findings. The body temperature of children with positive lung findings was higher than that of children with negative findings. Hospitalization time showed no statistical difference. We wondered whether inflammatory reaction was more obvious during younger children than older children. There were no statistical differences in duration of hospitalization or of viral shedding between the symptomatic and asymptomatic groups. The hospitalization period may be related to the government’s prevention and control program for the whole epidemic area during a specific period. All children with suspected or confirmed COVID-19 were admitted to our hospital for treatment. The discharge standard was in accordance with the unified requirements stipulated by the state. The length of a hospital stay not only considered the severity of the patient’s illness but also adhered to the discharge standard. This also suggested that most of the children had mild diseases and the hospitalization period itself was short. There were no differences in the median duration of viral shedding, which also indicated that there was no obvious correlation between viral load and clinical symptoms [21]. The viral load detected in asymptomatic populations was similar to that in symptomatic patients [16, 21]. Our duration of viral shedding was different from that reported by Lu et al. [6], who regarded the time from illness onset to discharge as the duration of viral shedding of SARS-CoV-2 in symptomatic children and from dates of last exposure or abnormal chest radiograph to discharge as the duration of viral shedding in asymptomatic children. We used the nucleic acid test results as the deciding criteria.

The limitations of this research are as follows: (1) The duration of viral shedding was based on the nasopharyngeal and throat swabs, confined only to the upper respiratory tract. We did not perform bronchoalveolar lavage so we do not know if the virus was present in the lower respiratory tract in children with lung involvement. (2) We did not analyze the link between viral load and the severity and prognosis of COVID-19. (3) Patients with more than one pathogen or diagnosis were excluded in this study.

## Conclusion

Mostly young children ≤3 years old were symptomatic, while children ≥6 years old were mostly asymptomatic. Respiratory symptoms are the main clinical manifestations, and the changes in laboratory indices were lower than those of adults. Fever was rarely observed. The average symptomatic temperature in this group was 37.2±1.1 °C. Most children were either asymptomatic or showed mild symptoms. The proportion of asymptomatic infection was higher and the probability of pneumonia was lower than that of the symptomatic group. Even if there are symptoms and lung abnormalities, the lesion size and involvement range are smaller than that in adults. Most lesions were absorbed and showed improvement after about 9 days.

## References

[1] Ai T, Yang Z, Hou H (2020). Correlation of chest CT and RT-PCR testing in coronavirus disease 2019 (COVID-19) in China: a report of 1014 cases. Radiology.

[2] Shi H, Han X, Jiang N (2020). Radiological findings from 81 patients with COVID-19 pneumonia in Wuhan, China: a descriptive study. Lancet Infect Dis.

[3] Ma H, Hu J, Tian J (2020). A single-center, retrospective study of COVID-19 features in children: a descriptive investigation. BMC Med.

[4] Xia W, Shao J, Guo Y (2020). Clinical and CT features in pediatric patients with COVID-19 infection: different points from adults. Pediatr Pulmonol.

[5] Hansell DM, Bankier AA, MacMahon H (2008). Fleischner society: glossary of terms for thoracic imaging. Radiology.

[6] Lu Y, Li Y, Deng W (2020). Symptomatic infection is associated with prolonged duration of viral shedding in mild coronavirus disease 2019: a retrospective study of 110 children in Wuhan. Pediatr Infect Dis J.

[7] Lu X, Zhang L, Du H (2020). SARS-CoV-2 infection in children. N Engl J Med.

[8] Guan WJ, Ni ZY, Hu Y (2020). Clinical characteristics of coronavirus disease 2019 in China. N Engl J Med.

[9] Sun P, Qie S, Liu Z (2020). Clinical characteristics of hospitalized patients with SARS-CoV-2 infection: a single arm meta-analysis. J Med Virol.

[10] Mehta NS, Mytton OT, Mullins EWS et al (2020) SARS-CoV-2 (COVID-19): what do we know about children? A systematic review. Clin Infect Dis. 10.1093/cid/ciaa556 10.1093/cid/ciaa556 PMC7239259 32392337

[11] Morand A, Fabre A, Minodier P (2020). COVID-19 virus and children: what do we know?. Arch Pediatr.

[12] Balasubramanian S, Rao NM, Goenka A (2020). Coronavirus disease 2019 (COVID-19) in children - what we know so far and what we do not. Indian Pediatr.

[13] Dong Y, Mo X, Hu Y (2020). Epidemiology of COVID-19 among children in China. Pediatrics.

[14] Arons MM, Hatfield KM, Reddy SC (2020). Presymptomatic SARS-CoV-2 infections and transmission in a skilled nursing facility. N Engl J Med.

[15] He D, Zhao S, Lin Q (2020). The relative transmissibility of asymptomatic COVID-19 infections among close contacts. Int J Infect Dis.

[16] Gao Z, Xu Y, Sun C et al (2020) A systematic review of asymptomatic infections with COVID-19. J Microbiol Immunol Infect. 10.1015/j.jmii.2020.05.001 10.1016/j.jmii.2020.05.001 PMC7227597 32425996

[17] Noh JY, Yoon JG, Seong H et al (2020) Asymptomatic infection and atypical manifestations of COVID-19: comparison of viral shedding duration. J Infect 21:S0163-4453(20)30310-8 10.1016/j.jinf.2020.05.035 PMC7240269 32445728

[18] Meng H, Xiong R, He R (2020). CT imaging and clinical course of asymptomatic cases with COVID-19 pneumonia at admission in Wuhan, China. J Inf Secur.

[19] Wang Y, Dong C, Hu Y (2020). Temporal changes of CT findings in 90 patients with COVID-19 pneumonia: a longitudinal study. Radiology.

[20] Perlman S (2020). Another decade, another coronavirus. N Engl J Med.

[21] Zou L, Ruan F, Huang M (2020). SARS-CoV-2 viral load in upper respiratory specimens of infected patients. N Engl J Med.

